# Mechanical, Electrical, and Thermal Properties of Carbon Nanotube Buckypapers/Epoxy Nanocomposites Produced by Oxidized and Epoxidized Nanotubes

**DOI:** 10.3390/ma13194308

**Published:** 2020-09-27

**Authors:** George Trakakis, Georgia Tomara, Vitaliy Datsyuk, Labrini Sygellou, Asterios Bakolas, Dimitrios Tasis, John Parthenios, Christoforos Krontiras, Stavroula Georga, Costas Galiotis, Kostas Papagelis

**Affiliations:** 1 Foundation of Research and Technology Hellas, Institute of Chemical Engineering Sciences (ICE-HT), P.O. Box 1414, GR-26504 Patras, Greece; trakakis@iceht.forth.gr (G.T.); sygellou@iceht.forth.gr (L.S.); jparthen@iceht.forth.gr (J.P.); c.galiotis@iceht.forth.gr (C.G.); 2 Department of Physics, University of Patras, 26504 Rio Patras, Greece; tomgeo@physics.upatras.gr (G.T.); krontira@physics.upatras.gr (C.K.); sgeorga@physics.upatras.gr (S.G.); 3 Physics Department, Institute of Experimental Physic, Free University Berlin, Arnimallee 14, 14195 Berlin, Germany; datsyuk@zedat.fu-berlin.de; 4 School of Chemical Engineering, National Technical University of Athens, GR-15773 Athens, Greece; abakolas@mail.ntua.gr; 5 Department of Chemistry (Section of Physical Chemistry), University of Ioannina, 45110 Ioannina, Greece; dtassis@uoi.gr; 6 Department of Chemical Engineering, University of Patras, GR-26504 Patras, Greece; 7 School of Physics, Department of Solid State Physics, Aristotle University of Thessaloniki, 54124 Thessaloniki, Greece

**Keywords:** buckypaper, nanocomposites, mechanical properties, electrical properties, thermal properties

## Abstract

High volume fraction carbon nanotube (CNT) composites (7.5–16% vol.) were fabricated by the impregnation of CNT buckypapers into epoxy resin. To enhance the interfacial reaction with the epoxy resin, the CNTs were modified by two different treatments, namely, an epoxidation treatment and a chemical oxidation. The chemical treatment was found to result in CNT length severance and to affect the porosity of the buckypapers, having an important impact on the physico-mechanical properties of the nanocomposites. Overall, the mechanical, electrical, and thermal properties of the impregnated buckypapers were found to be superior of the neat epoxy resin, offering an attractive combination of mechanical, electrical, and thermal properties for multifunctional composites.

## 1. Introduction

Graphitic nanostructures have attracted great scientific and industrial attention recently due to their exceptional mechanical, electrical, and thermal properties [1]. As is evident, graphitic nano-inclusions, such as CNTs and graphene, have to be incorporated into matrices in order to be used in various applications. This requires adequate interface interactions between the inclusion and the matrix in order to exploit the extraordinary physical and mechanical properties of graphitic materials.

Another challenge relates to adequate processing methods for efficient impregnation of the nano-inclusions. For one-dimensional filler nanomaterials, one such method is to first form dry paper-like assemblies of such nanostructures, similar to carbon fiber fabrics that are used as reinforcing media in composite applications. 

Buckypapers are thin sheets of highly porous and randomly entangled CNT networks. The most common preparation method is the vacuum filtration of well-dispersed CNT suspensions [2]. The inherent properties (e.g., thermal and electrical conductivity) and structure (e.g., porosity) of the unmodified buckypapers can be affected by various factors, such as the type of CNT used, their chemical modification etc. [2,3,4,5]. As it is well known, CNTs are nanomaterials with extremely high aspect ratios and they tend to agglomerate, forming bundles. The buckypapers are, in fact, a usable macroscopic form of CNT assemblies, with the aim to overcome agglomeration phenomena and finally to take advantage of the exceptional properties of individual CNTs [6,7,8]. 

Buckypapers have been extensively used in a variety of applications as filters [9], fire retardancy materials [10], electromagnetic shielding materials [11,12], catalyst supports [13,14], actuators [15,16], sensors [17,18], supercapacitors [19,20], and fuel cell electrodes [21,22]. They have even been used in medical applications [23], but probably the most widespread usage of them is the incorporation into polymeric matrices for the enhancement of their mechanical, electrical, or thermal properties [7,8,24,25,26].

Progress in buckypaper/polymer composites has been extensively recorded in the literature [27,28]. These hybrid materials offer a great variety of properties, able to strengthen existing multifunctional applications or conjure new ones. The properties of new materials are strongly related to many parameters, some of them are still unknown or not studied well. By exploring and tuning these parameters upgraded properties can be achieved. This also contributes to “mold” all the knowledge from an enormous number of experimental observations and conduce to general remarks about structure-properties relationship of buckypapers/composites or, even broadly, of all kinds of nanocomposites.

From previous work of our group related to composites, and especially nanocomposites [2,7,8,21,29,30,31,32,33,34], we have concluded that the most important parameters in buckypaper-composites are quality of buckypaper impregnation (buckypaper porosity), CNT-matrix interphase, length of CNTs, orientation of CNTs, dispersion of CNTs into matrix, fraction of CNTs in final composites, and the viscosity of matrices. Some of these parameters are strongly correlated by each other [7,8]. Tuning of these parameters may dramatically increase the mechanical properties of buckypaper/nanocomposites [7,8]. In this work we continue the study of the final mechanical, electrical, and thermal properties of these nanocomposites. More specifically, we present here the characterization of oxidized CNTs and buckypapers and discuss the differences between them and epoxidized ones, previously reported [7]. The results gave prominence to the crucial role of buckypapers porosity, and mostly, revealed that the buckypaper approach can offer composite materials with an attractive combination of properties for a wide range of applications, such as supercapacitors, sensors, flexible structures [29], structural materials with EMI shielding abilities, energy storage, cooling elements, automotive, and aeronautics applications, etc. [35].

## 2. Materials and Methods

The nanocomposite materials produced following four individual procedures. Firstly, the surface of CNTs was chemically modified. Secondly, the modified CNTs were used to form buckypapers. As a third step, buckypapers were immersed into an epoxy/hardener mixture followed by refrigeration for prepregs formation. Finally, the prepregs were laminated by autoclave processing for nanocomposites fabrication.

### 2.1. Materials

MWCNTs (NC3100, ~15.5 μm length and 9.5 nm diameter) were supplied by Nanocyl (Sambreville, Belgium). Sulfuric acid (H_2_SO_4_, 258105 Sigma-Aldrich, Munich, Germany), dichloromethane (CH_2_Cl_2_, 32222 Honeywell, Charlotte, NC, USA), potassium permanganate (KMnO_4_, 13206 Sigma-Aldrich), 3-chloroperoxybenzoic acid (255795000 Acros Organics, Geel, Belgium) were used for the chemical treatment. For nanocomposites production, Sicomin^®^ SR 1700 epoxy monomer/Sicomin^®^ SD 2803 curing agent (Chateauneuf les Martigues, France) were used.

### 2.2. Chemical Functionalization of CNTs

Two different chemical routes were tested for the chemical modification of the outer surface of CNTs. Firstly, an epoxidation treatment for the addition of epoxy rings on CNTs [36]. Hydroxyls/carboxyls groups are also present. Secondly, an oxidation reaction adding various chemical groups containing oxygen (carboxyls, carbonyls, hydroxyls) on CNTs [37]. Additionally, for both routes three approaches were investigated to adjust the grafting ratio of the functional groups; an aggressive chemical reaction to add many chemical groups on CNTs, an intermediate and a mild reaction. Finally, six kinds of CNTs were available: Highly grafted epoxidized CNTs (for the sake of convenience they are named as “HG-E-MWCNTs”), moderately grafted epoxidized CNTs (MG-E-MWCNTs), slightly grafted epoxidized CNTs (SG-E-MWCNTs), highly grafted oxidized CNTs (HG-O-MWCNTs), moderately grafted oxidized CNTs (MG-O-MWCNTs), and slightly grafted oxidized CNTs (SG-O-MWCNTs).

Regarding epoxidation treatment, for the first batch, 7 g of epoxidation reagent (3-chloroperoxybenzoic acid) was dissolved in 170 mL CH_2_Cl_2_. Then, 1.75 g of MWCNTs was added and the solution was stirred for about 20 h at room temperature. The reaction mixture was filtered through a 0.2 μm PTFE membrane filter and washed with excess CH_2_Cl_2_. The epoxidized CNTs were redispersed twice in CH_2_Cl_2_ by magnetic stirring, filtered and dried under vacuum at 80 °C. To decrease the grafting ratio onto the CNT sidewalls, a lower concentration of epoxidizing agent was used; 1.75 g of 3-chloroperoxybenzoic acid were mixed with 1.75 g of nanotubes in 170 mL CH_2_Cl_2_, and the mixture was stirred for a period of 20 h. The third protocol involved the very same concentration as the previous, but for a period of 15 min [7]. 

Concerning the oxidation protocol, for a high degree of grafting ratio, 2.6 g of multi-walled CNTs were dispersed in 260 mL of 0.5 M sulfuric acid by ultrasonic vibration for 5 min in a flask. The suspension was refluxed in an oil bath at 120 °C with magnetic stirring. Meanwhile, 25 g of KMnO_4_ was dissolved in 260 g of 0.5 M sulfuric acid, and this solution was added to the flask dropwise. The mixture was kept at 120 °C for 3 h. After that period, the resulting suspension was filtered, washed with hydrochloric acid and deionized water and then dried. These oxidized CNTs were named “HG-O-MWCNTs”. For medium grafting ratio the oxidation protocol was repeated using 5 g of KMnO_4_ and the mixture was kept for 15 min (MG-O-MWCNTs) and, finally, for an even lighter functionalization 1.75 g of KMnO_4_ and 1.75 g of CNTs were mixed for 15 min (SG-O-MWCNTs).

### 2.3. Buckypapers Production

Buckypapers were produced by the following method: firstly 250 mg of MWCNTs were dispersed in H2O to prepare stable CNTs solutions of 1 mg/mL, by tip sonication for 15 min. The suspensions were then filtered by vacuum filtration through polycarbonate membranes of 0.4 μm pore size. The drying was performed by hot air and then the formed buckypapers were peeled off from the filter. The average thickness of the buckypapers was about 130–230 μm, depending on the type of functionalized CNTs, while their diameter was about 7 cm.

### 2.4. Prepregs and Nanocomposites Production

To form prepregs, an immersion was performed for a minute of buckypapers into a low viscosity solution of Sicomin^®^ SR 1700 epoxy monomer/Sicomin^®^ SD 2803 curing agent mixture (100:39 mass ratio). The temperature was 40 °C. After the soaking, the resin-filled buckypapers were extracted from the mixture and were refrigerated to −18 °C for prepregs formation. 

Regarding nanocomposites production, the prepregs were laminated by an Aeroform^®^ autoclave (Dorset, UK) for curing. Curing conditions were 24 h at 28 °C under a pressure of 6 atm, and for post curing, 8 h at 80 °C. For each nanocomposite, eight prepregs were used. The volume fraction of CNTs was estimated to be 16% for HG-O-nanocomposite, 14% for MG-O-nanocomposite, and 11% for SG-O-nanocomposite.

### 2.5. Characterization

To determine the result of CNTs functionalization by terms of mass change, thermogravimetric analysis (TGA) measurements were carried out. Modified and unmodified CNTs were heated to 680 °C, by a rate of 10 °C/min, in Nitrogen atmosphere. The equipment for the analysis was TA Q50 (New Castle, DE, USA). The details of pores of the dry (empty) buckypapers were studied by mercury intrusion porosimetry (Thermo Electron Corp., Waltham, MA, USA, Porosimeter Pascal 440). The architecture of the internal CNT network and the penetration quality of epoxy were explored by SEM (LEO SUPRA 35 VP, Carl Zeiss, Oberkochen, Germany). Individual CNTs were examined by a JEM-2100 TEM (Jeol, Tokyo, Japan). Tensile properties of the neat buckypapers were studied by a TA Q800 instrument (New Castle, DE, USA, displacement rate: 500 μm/min, 5 strips of 30 mm × 4 mm in dimension, for each film type). The surface modification of CNTs were studied by XPS in a UHV chamber equipped with a SPECS LHS-10 hemispherical electron analyzer (Berlin, Germany) [7]. The mechanical properties of nanocomposites and pure resin were studied by three-point bending experiments. A Hounsfield machine (Surrey, UK) was used. 5 strips were tested for each type of material in accordance to ASTM D790. For electrical conductivity measurements, broadband dielectric measurements were performed, using an Alpha-N frequency response analyzer by Novocontrol Technologies GmbH (Hundsangen, Germany) [34]. The thickness of the samples was between 1.5 and 2 mm, while the diameter of the specimens was 30 mm. Finally, the thermal conductivity of our samples was measured using a HotDisk TPS 2500 S transient plane source (HotDisk AB, Gothenburg, Sweden) [38]. The measurements were performed at 23 °C. 

## 3. Results and Discussion

### 3.1. TEM of Individual CNTs

**Figure 1:**
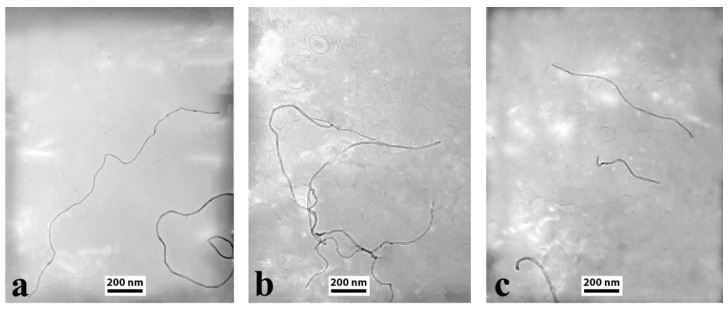
TEM images of (**a**) unmodified CNTs, (**b**) HG-E-MWCNTs, and (**c**) HG-O-MWCNTs.

To clarify the effect of the different chemical modification routes on the structure of CNTs, individual CNTs from unmodified, HG-E and HG-O batches were observed by TEM (Figure 1). More specifically, the length of ~50 individual CNTs was measured and the average length was estimated. The length of unmodified CNTs was found ~1.5 μm, in accordance to Nanocyl’s specifications. Epoxidized CNTs were also found ~1.5 μm in average, which proves that the epoxidation treatment is a non-destructive functionalization of CNTs. On the other hand, the length of oxidized CNTs was found much shorter (~600 nm), indicating a high degree of CNTs severance. It has been previously reported in the literature that exposure of CNTs into oxidative media affects significantly the structural integrity of CNTs [39,40]. TEM observations are very important as the length of CNTs is a key parameter for properties of buckypapers and nanocomposites.

### 3.2. SEM Study of Neat Buckypapers

**Figure 2:**
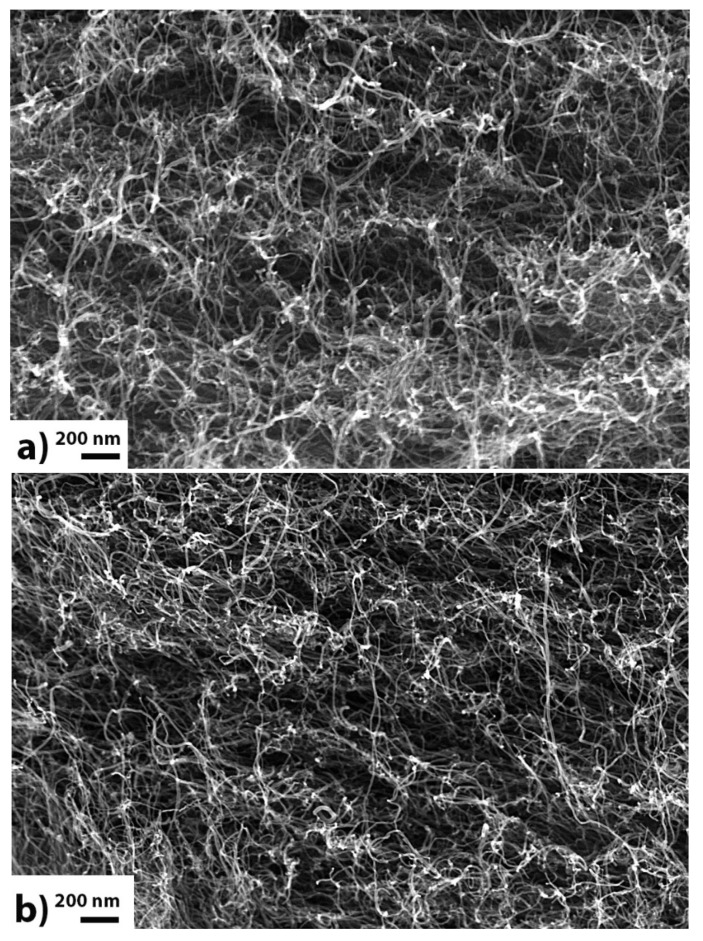
Cross-section of an HG-E (**a**) and an HG-O (**b**) buckypaper (SEM capture).

The topological architecture of the buckypapers was studied by SEM imaging. In Figure 2, typical SEM images of a SG-E-MWCNTs (**a**) and a SG-O-MWCNTs buckypaper (**b**) are presented. The films consists of individual and randomly-oriented CNTs. Their porous character is obvious and is common to all types of buckypapers, produced in this and previous work [7]. Macroscopically, no structural differences between the two types are observed.

### 3.3. Thermogravimetric Study

**Figure 3:**
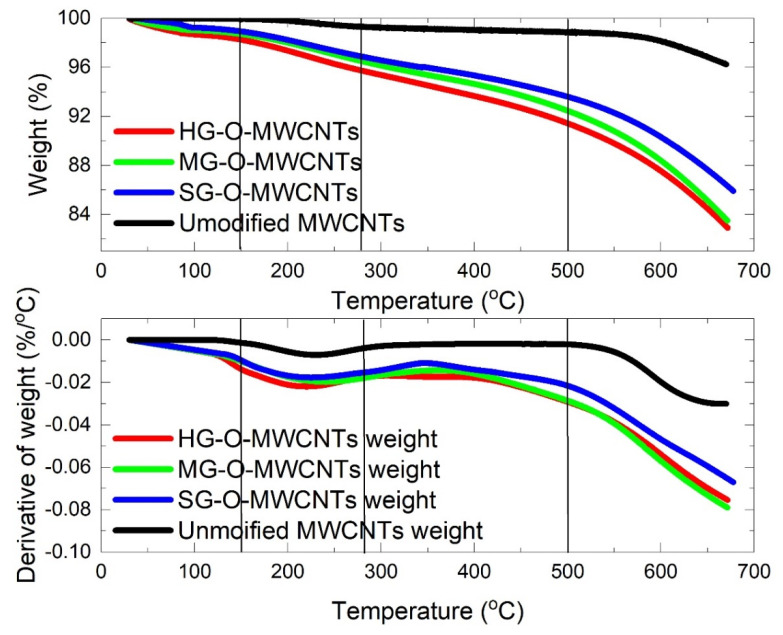
TGA analysis of unmodified and oxidized CNTs.

Figure 3 presents TGA and DTG curves of the oxidized (HG-O-MWCNTs, MG-O-MWCNTs, and SG-O-MWCNTs) and unmodified CNTs. The data and analysis for epoxidized CNTs have been reported previously [7], while detailed mass losses of the oxidized and epoxidized CNTs are shown in Table 1. There is a difference between the weight loss curves of unmodified and oxidized CNTs. The different behavior can be explained by the addition of chemical groups on CNTs surface chemical modification. The X axis of temperature can be divided into four sections [7]; weight loss between 30 and 150 °C is attributed to evaporation of physically absorbed solvent traces that have been originated in the functionalization/filtration processes. The next temperature window (150–280 °C) is assigned to decarbonylation and decarboxylation from labile groups of MWNTs sidewalls and tips, resulting in elimination of either CO or CO_2_ gases [41]. Such elimination processes may potentially take place at temperatures up to about 350 °C [40]. In the third region (280–500 °C) the thermal degradation is explained by the elimination of covalently attached epoxy/hydroxy groups onto the CNT surface. At temperatures above 500 °C, the observed weight loss corresponds to the thermal pyrolysis of defected carbon atoms onto the graphitic lattice, having sp^3^ hybridization [40]. Regarding Table 1, there is an evident difference between the unmodified and the modified CNTs, too. This observation indicates the successful grafting of chemical groups at the surface of the CNTs. Moreover, small differences between the three batches for each treatment can be observed. By the summation of the weight losses from the second and the third stage (due to elimination of functionalities, see [7]), we have an indication that the different combination of concentration/duration treatment has led to very little, yet, different grafting ratios, as the HG-O protocol yielded 6.89 wt % functionalities, the MG-O 6.23 wt % and finally the SG-O protocol about 5.33 wt %.

### 3.4. XPS Characterization

**Figure 4:**
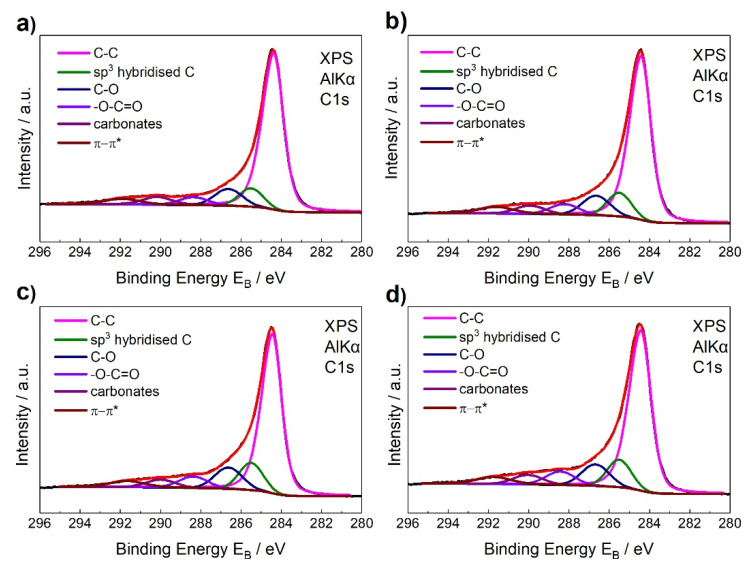
Deconvolution of the C1s peaks for (**a**) unmodified, (**b**) SG-O-MWCNTs, (**c**) MG-O-MWCNTs, and (**d**) HG-O-MWCNTs.

**Figure 5:**
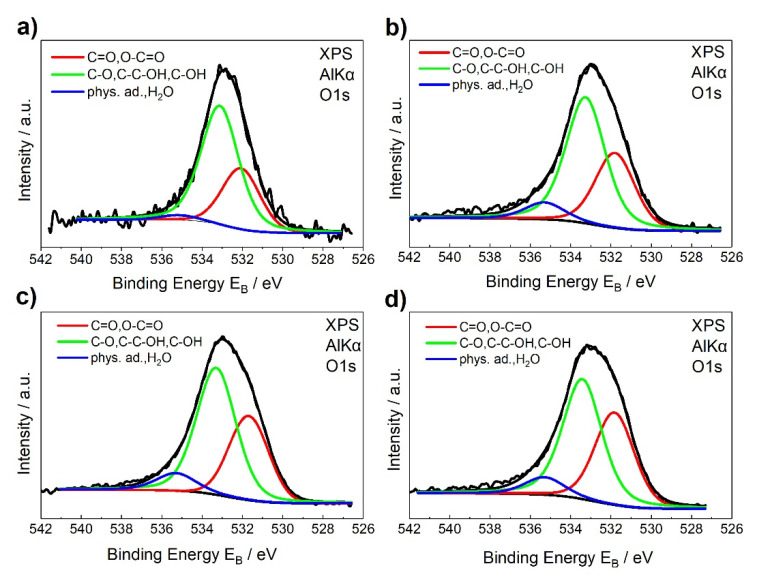
Deconvolution of the O1s peaks for (**a**) unmodified, (**b**) SG-O-MWCNTs, (**c**) MG-O-MWCNTs, and (**d**) HG-O-MWCNTs.

XPS characterization is a very efficient method to study the chemical identity of surfaces. In current study, it was used to determine the chemical species of groups grafted/adsorbed on the CNTs surface and the density of defects that are present on its lattice. In Figure 4 and Figure 5 the C1s and O1s peaks of the unmodified and HG-O-MWCNTs, respectively, are shown (for epoxidized CNTS, see [7]). Deconvolution of C1s peak revealed total six peaks, which can be assigned to specific chemical groups [39,40]. O1s spectra (Figure 5) deconvoluted into three components [40], strengthened the efficient grafting with oxygen-containing groups.

Table 2 presents extracted data from the C1s and O1s spectra for all kind of used CNTs and they lead to some very useful conclusions. Firstly, they confirmed the successful decoration with oxygen-containing moieties during oxidation treatment. With the total oxygen content being up to 10.6% in the sample denoted as HG-O. From the values of % total oxygen contents, it is quite clear that concentration of oxidizing agent and duration of oxidation reaction seem to be crucial parameters for the grafting density of oxygenated groups, as it was also observed for epoxidation treatment [7]. More specifically, the total oxygen content from the CNTs surface increased from 2.5% for unmodified CNTs to 7.7, 10.4, and 10.6% for SG-O, MG-O, and HG-O, respectively.

The XPS analysis also showed an increase at the percentage of defected carbon atoms on the graphitic structure (sp^3^/sp^2^ ratios), as the oxidation treatment becomes more violent. More defects are observed at HG-O CNTs, where sp^3^/sp^2^ ratio is 0.16. 

### 3.5. Porosimetry Analysis

**Figure 6:**
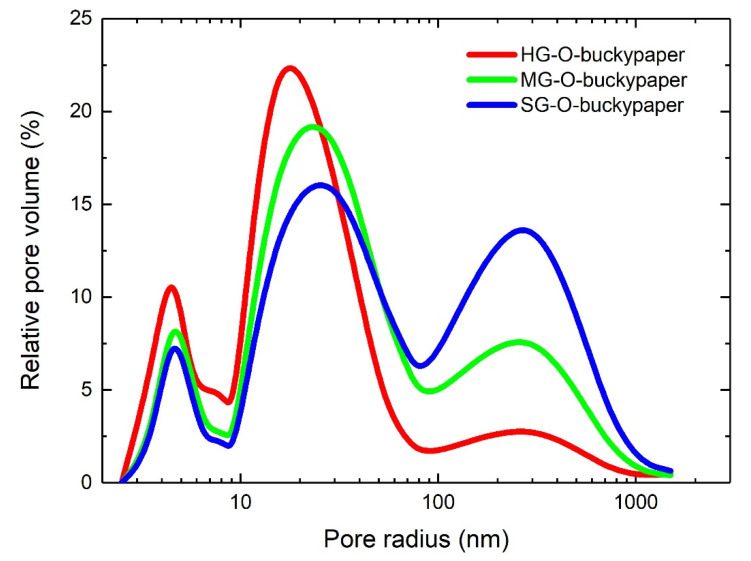
Pore size distribution from mercury porosimetry of oxidized buckypapers.

To explore the internal structure of the dry (neat) buckypapers, porosimetry measurements were performed. The pore size is a crucial parameter to produce high volume fraction composites, since small pores block the impregnation of the resin molecules inside the empty space of the buckypaper, leaving empty holes which act like defects [25]. In Figure 6 and Table 3 the percentage of pore volume as a function of pore size and the characteristic values as determined by porosimetry of the produced buckypapers are presented. In Table 3 values for epoxidized samples have been introduced, too. Three main pore distributions are detected as it was shown previously in epoxidized buckypapers [7], but the relative pore volume for each peak is different. Here, the more aggressive the oxidation the smaller the pores sizes are. This effect can be attributed to the severance of CNTs, as the high oxidation cuts the CNTs into smaller tubes which are packed more efficiently during buckypaper formation, driving into smaller pores. This is reflected also to the total porosity of the buckypapers: the HG-O-buckypaper has a total porosity of 39%, the porosity of the MG-O-buckypaper is 53% and the porosity of the SG-O-buckypaper is 61%. This is even more clear taking into account the average pore radius: 12 nm for HG-O, 22 nm for MG-O and 31 nm for SG-O. Thus, the length of the tubes seems to be a crucial parameter that governs buckypapers porosity, while in our previous work, where all functionalized CNTs had the same length, the grafting density of functional groups on the surface of the CNTs defined the porosity [11]. Additionally, it must be noticed that epoxidized buckypapers are more porous (69–74%) with larger pore radius (48–69 nm).

### 3.6. Tensile Experiments of Buckypapers

**Figure 7:**
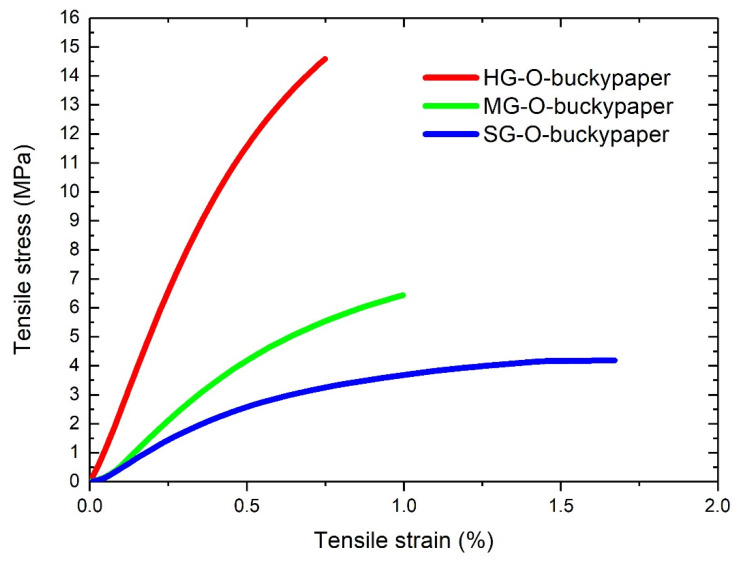
Stress-strain curves of buckypapers.

In Figure 7 stress-strain curves of oxidized, neat (without resin) buckypapers are presented, generated by tensile experiments, while Table 4 presents the engineering values extracted from these curves and also from epoxidized samples. The HG-O-buckypaper is the most brittle, reaching an ultimate strength of 14 MPa and a Young modulus of about 3 GPa. As the oxidation treatment becomes gentler, the strength and the modulus get downgraded. This behavior is correlated to the porosity of the buckypapers, namely, as the HG-O-buckypapers have the smaller porosity, the number of CNTs contacts is higher and the friction forces are increased, resulting to higher strength and modulus. Due to their denser structure, oxidized buckypapers have strength and modulus higher than the epoxidized. For a detailed analysis regarding the porous character of buckypapers and their mechanical properties please see our previous work [7].

### 3.7. SEM of Oxidized Nanocomposite Materials

**Figure 8:**
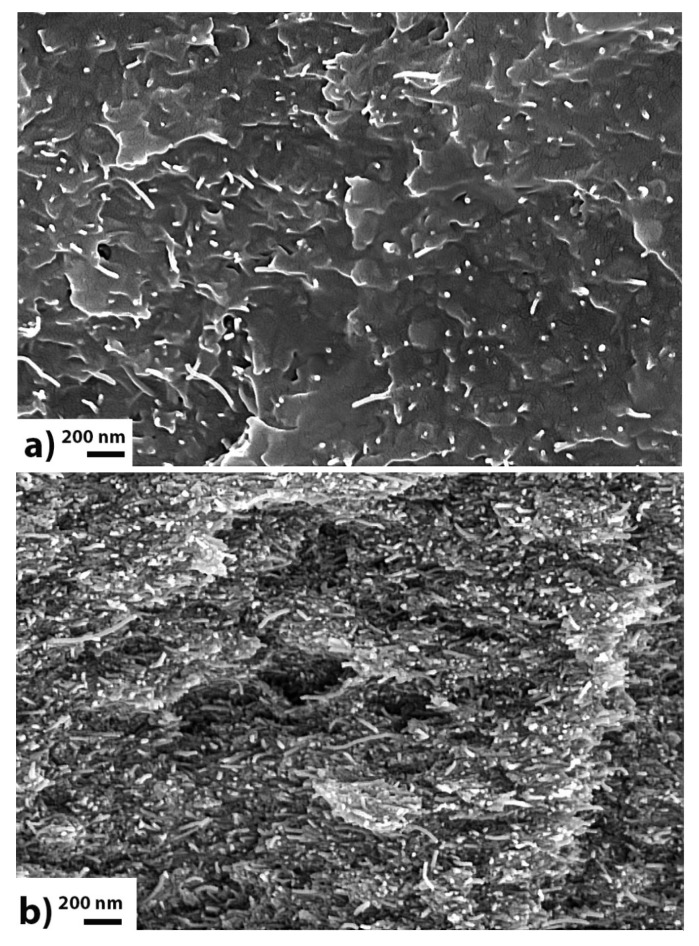
SEM capture from the cross-section of the HG-E (**a**) and HG-O (**b**) nanocomposites.

The evaluation of CNTs dispersion and resin impregnation quality was performed by SEM photos from the cross-section of the nanocomposites. Figure 8 presents such photos from HG-E and HG-O-nanocomposites. It is clear that a very homogenous dispersion has been achieved for both materials. The resin has impregnated the entire structure of HG-E due to the large porosity of buckypapers, while HG-O seems to have some empty pores. A noticeable point at HG-O is the presence of some pulled-out CNTs, indicating perhaps a weak adhesion between the polymer and CNTs locally.

### 3.8. Flexural Properties of Nanocomposite Materials

**Figure 9:**
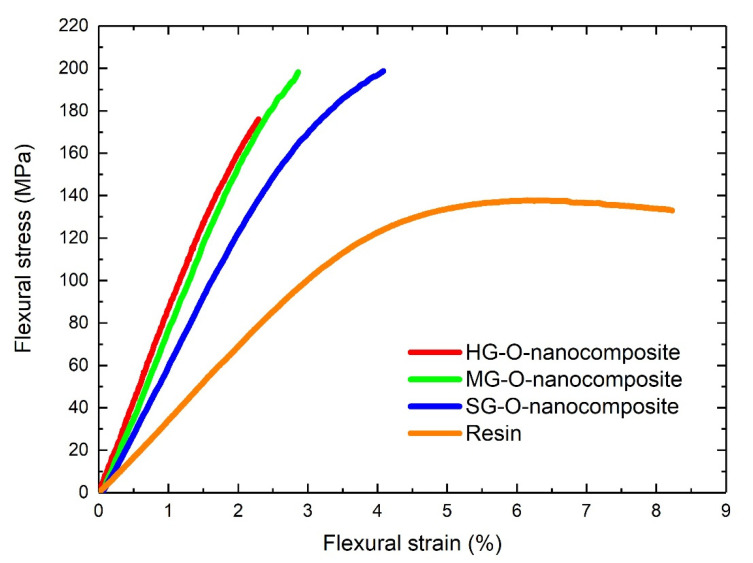
Bending properties of oxidized nanocomposites and neat resin.

To investigate the mechanical properties of the produced nanocomposites, three-point bending experiments were performed. In Figure 9 the flexural behavior of the tested materials are shown, while in Table 5 the extracted engineering values from the bending tests, including epoxidized samples, are shown. It is evident that successful reinforcement of the resin has achieved for all nanocomposites. For oxidized samples, the highest flexural modulus was recorded for HG-O-nanocomposite (~8.4 GPa), due to the highest volume fraction of CNTs (16%). This modulus is increased 144% in comparison with the modulus of resin. In addition, the strength of HG-O-nanocomposite is lower than the modulus of the other two nanocomposites. This effect can be explained by two factors: (a) the smaller porosity of the HG-O-buckypapers: the resin cannot fill the entire structure of the films, so there are empty pores which act like defects, downgrading the strength which is an engineering value more sensitive to imperfections. (b) the length of the HG-O-MWCNTs: as it has been mentioned, the strong oxidation treatment cuts the CNTs into smaller tubes. As the strength at composite materials is highly corelated to the length of reinforcement [7], the strength of HG-O-nanocomposites are lower than the other two. The best flexural strength was recorded by SG-O-nanocomposite (203 MPa, 50% increase compared to resin). This material was produced by SG-O-buckypaper, which had a large porosity, allowing to the resin to completely impregnate the CNTs, without empty spaces. The volume fraction of this nanocomposite was estimated 11%. Regarding epoxidized nanocomposites [7] the best sample presented a modulus of 6.63 GPa, and a strength of 232 MPa (72% increase). For a comprehensive analysis regarding the connection between the porosity of buckypapers, the length of CNTs, and the mechanical properties of nanocomposites see [7].

As a general conclusion from our findings from our previous [7] and this work regarding mechanical properties of polymer nanocomposites produced by CNTs buckypapers the ideal porosity of the thin sheets should offer the optimum compromise between large pore diameters for resin to completely impregnate the CNTs, and small ones for high CNTs volume fractions. Additionally, between the two different chemical modifications we tested, it seems that epoxidation offers better bonding between resin and CNTs, hence, better stress transfer and mechanical properties.

Moreover, it is worth noticing that the observed flexural strength is comparable with glass fiber epoxy composites [42].

### 3.9. Electrical Performance of Nanocomposites

**Figure 10:**
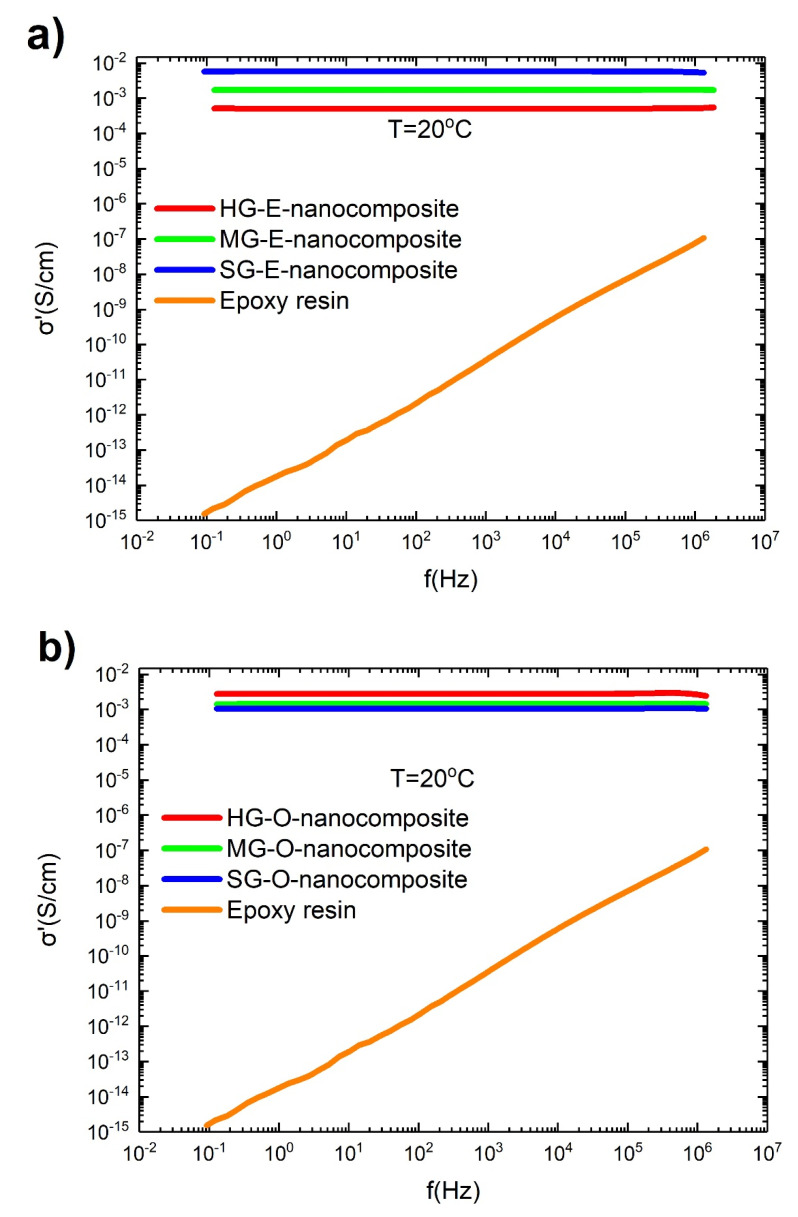
σ’ conductivity as a function of frequency at 20 °C for epoxy resin and (**a**) epoxidized nanocomposites, (**b**) oxidized nanocomposites.

All nanocomposites were tested for electrical conductivity at 20 °C by broadband electrical spectroscopy. Figure 10 present the electrical conductivity measurements as a function of frequency (0.1 Hz–1 MHz) for nanocomposites and epoxy resin. Table 6 presents the values of conductivity at 0.1 Hz, which, for percolated materials, corresponds to DC conductivity [34]. As is well known, the results reveal that the epoxy resin is an electrical insulator, as its DC conductivity is 1.5 × 10^−15^ S/cm. Its conductivity is also strongly dependent on frequency, but even at 1 MHz it is about 10^−7^ S/cm. DC conductivity (σ’ at 0.1 Hz) of both epoxidized and oxidized nanocomposites is much higher than resin at 0.1 Hz, 10^−3^ S/cm, evident that the addition of CNTs increases many orders of magnitude the electrical conductivity of resin (Figure 10, Table 6). An interesting point is that conductivity is independent of frequency. 

It is well established that when the concentration of CNTs in nanocomposites overreaches a critical value, then the electrical behavior of the material is changed, and from insulator becomes conductor [34]. This critical value is named percolation threshold. When percolation threshold has been overpassed, then the conductivity of the material is increased many orders of magnitude. This happens because a conductive network from CNTs is formed. CNTs are in contact or at very close distances, so that electrons are able to transport through CNTs by crossing over each other, or by tunneling effect [43]. The formation of this CNTs network, or in other words, the formation of a network of conductive paths is the basic mechanism for electrical conductivity in CNT/polymers composites [44,45]. Seidel et al. [46] developed a micromechanical model describing this conductivity effect in such networks, while other researchers contributed to the understanding of this phenomenon [31,33,47,48,49]. In many works at literature the percolation threshold has been calculated for CNTs/polymers composites [31,32,50,51] and it is proven that a very small amount, usually below 1% vol., is enough to covert dielectric polymers to conductive materials. Higher concentration can even decrease the conductivity, due to ineffective dispersion of CNTs and bundles formation [52].

Taking in account the above considerations, the experimental results of electrical measurements can easily be explained. The behavior of resin (Figure 10) is identical to dielectric materials, as conductivity increases with frequency. In addition, conductivity of nanocomposites is independent from frequency, forming a plateau. This plateau is characteristic of a conductive network formation [31], and it proves that in these specific nanocomposites percolation threshold has been overreached.

The differences between electrically conductive behavior of nanocomposites (Figure 10) are not in fact a matter of grafting but exist mostly due to different CNTs concentrations. For oxidation case, the high oxidation treatment cuts the CNTs into smaller tubes, this leads to smaller porosity of buckypapers, and this also leads to higher concentration of CNTs, which means higher conductivity. Thus, an increase in conductivity is observed as concentration is also increased, as more conductive paths are created [53]. Additionally, epoxidized nanocomposites present a slightly higher conductivity compared to oxidized, because of better CNTs dispersion into matrix and longer CNTs. Length of CNTs have and important effect on total conductivity of nanocomposites [54,55]. 

It should be mentioned that the observed electrical conductivities are comparable with those of semiconductors [56].

### 3.10. Thermal Performance of Nanocomposites

**Figure 11:**
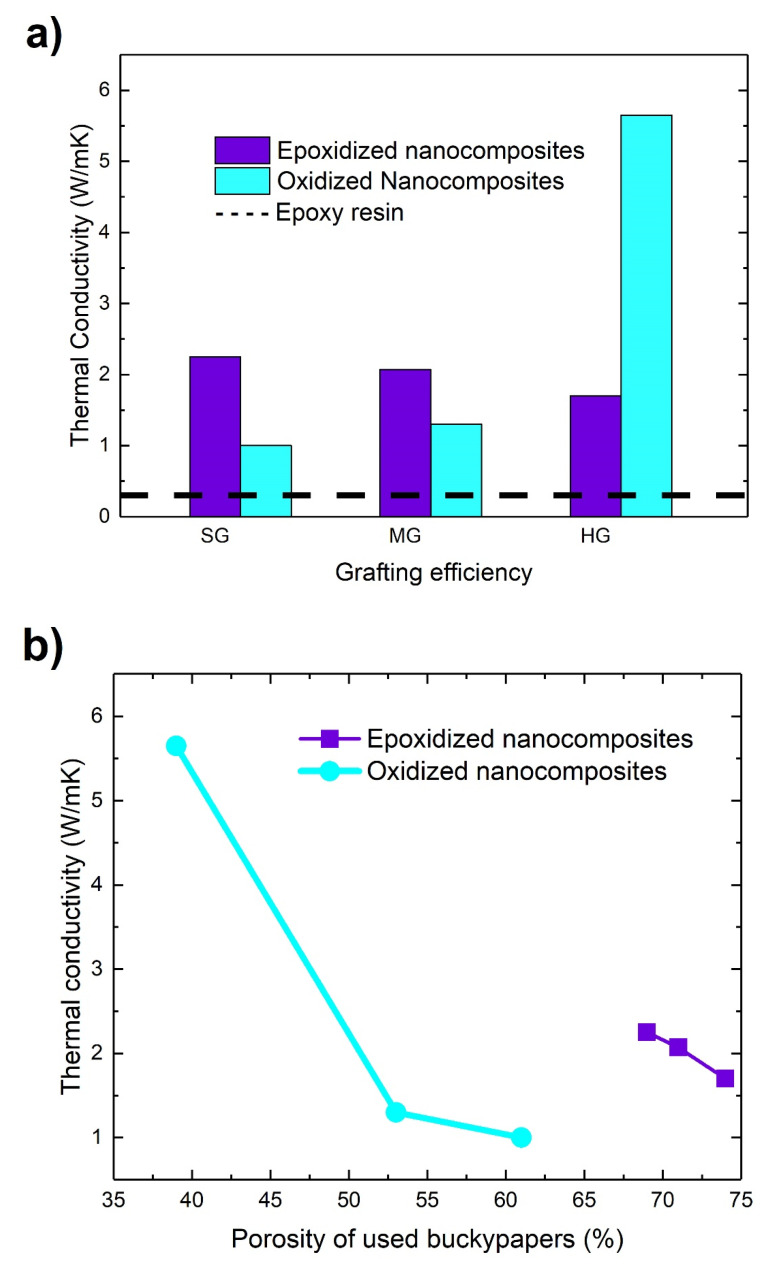
(**a**) Thermal conductivity of nanocomposites with their grafting efficiency, (**b**) thermal conductivity as a function of porosity of used buckypapers.

The thermal conductivities of epoxy resin and carbon nanotube buckypaper-polymer nanocomposites measured by HotDisc technique are presented in Figure 11a. The epoxy resin has a thermal conductivity of 0.29 W/mK, while all the studied nanocomposites exhibit higher values. Thus, in epoxidized samples, the SG-E-nanocomposite has the higher conductivity between them (2.25 W/mK, 675% increase compared to resin). In oxidized samples the HG-O-nanocomposite produced by the smaller porosity buckypaper, has the higher conductivity (5.65 W/mK, increased ~1850% compared to resin). From a first point of view it seems that the treatments affect the conductivity (Figure 11a), but the main affecting factor is the porosity of the used buckypapers (which depends on the treatment), as Figure 11b shows. Generally, as the porosity of the used buckypapers is decreased, the thermal conductivity increases.

Due to the extremely high thermal conductivity of the CNTs (~3000 W/mK for Nanocyl 3100 MWCNTs) many researchers had proposed that CNTs is the desired thermal reinforcing material for polymers, by the formation of conducting CNTs networks [57,58,59,60]. However, the experimental results revealed that this expectation was far away from realization. Indeed, in thermal conductivity measurements although the conductivity was increased, it was not comparable with CNTs conductivity values [61,62]. Additionally, a large deviation was observed in results, from important reinforcement by a very small amount of CNTs [60], to downgrading the thermal conductivity [63]. The above reveal the complication of the effect of thermal reinforcement of polymers by CNTs and the dependence by many factors. The thermal conductivity of composite materials and the parameters that affect it have been nicely reviewed by Burger et al. [64]. Some of these parameters include defects, phonon scattering, type of filler, structure, functionalization and alignment of them, and network formation.

Taking into account the above factors that affect the thermal conductivity of nanocomposites, some explanation of the observed results can be proposed. Buckypapers act like a scaffold for a thermal conductive network. In that case, CNTs are the bridges on which phonons pass through the resin, without important energy losses. Of course, losses in CNT-CNT junctions still exist, but they are not so important as the phonon scattering in CNT-resin interfaces. Additionally, the network ensures the absence of bundles. When bundles are present significant volume fraction of nanocomposite is empty from CNTs, generating areas with very low thermal conductivity (neat resin) and areas with high thermal conductivity (CNTs bundles). As a result, the overall conductivity of the nanocomposite is problematic. Thus, the smaller porosity of buckypapers and the higher volume fraction of CNTs in nanocomposites is desirable for high thermal conductivity as they offer more bridges to phonons for sufficient conduction.

From the results of this work it is obvious that the addition of CNTs in epoxy resin by the buckypapers approach enhances considerably the thermal conductivity. For example, HG-O-nanocomposite has a conductivity which is ~1850% higher than conductivity of matrix. The improvement is due to the presence of conductive paths by CNTs inside resin, originating from buckypapers structure. Thus, the proposed method is proven to be very efficient for the thermal property improvement of polymers. In fact, the observed conductivities of this work are amongst the higher values in the literature for polymer nanocomposites [65,66,67].

## 4. Conclusions

In this work, CNTs/epoxy nanocomposites were produced and studied. The production was performed by the buckypaper approach: firstly, CNTs were chemically modified and formed dry thin films by vacuum filtration. Buckypapers were used to produce prepregs and final nanocomposites. It was proven, that this method is very efficient for the enhancement of matrix material’s properties. More specifically, CNT buckypapers can convert epoxy resin from low mechanical performance material to high performance comparable to glass fiber/epoxy composites, from electrical insulator to semiconductor and from thermal insulator to thermal conductor. As prepregs have become the main raw materials in composites industry, the proposed method of this work could easily be used by composites science and technology for new materials with tailored combined mechanical, electrical, and thermal properties.

## Figures and Tables

**Table 1:** Weight losses of unmodified, oxidized and epoxidized [7] CNTs from TGA.

Material	30–150 °C	150–280 °C	280–500 °C	500–680 °C	Total	Total of 150–500 °C (Functionalities)
	Weight Loss (%)					
Unmodified CNTs	0	0.70	0.50	2.83	4.03	1.2
SG-O-MWCNTs	1.07	2.07	3.26	9.17	15.57	5.33
MG-O-MWCNTs	1.29	2.23	4.00	10.66	18.16	6.23
HG-O-MWCNTs	1.75	2.53	4.36	11.33	19.97	6.89
SG-E-MWCNTs	0.78	4.02	1.81	4.80	11.41	5.83
MG-E-MWCNTs	0.92	4.14	1.81	5.10	11.97	5.95
HG-E-MWCNTs	0.65	4.46	1.82	6.14	13.07	6.28

**Table 2:** Extracted data from deconvolution of C1s and O1s peaks for unmodified, oxidized, and epoxidized [7] CNTs.

CNTs	Total Oxygen Amount (%)	Carbonyls/ Carboxyls (%)	Hydroxyls/ Epoxides (%)	Physically Adsorbed Oxygen and H_2_O Content (%)	sp^3^/sp^2^
Unmodified	2.5	0.78	1.66	0.06	0.10
SG-O-MWCNTs	7.7	2.52	4.56	0.61	0.12
MG-O-MWCNTs	10.4	4.56	5.19	0.65	0.14
HG-O-MWCNTs	10.6	4.04	5.72	0.84	0.16
SG-E-MWCNTs	4.4	1.72	2.40	0.28	0.12
MG-E-MWCNTs	8.5	3.47	4.14	0.88	0.13
HG-E-MWCNTs	12.3	7.25	4.62	0.42	0.15

**Table 3:** Porosimetry data extracted from porosimetry curves of neat, oxidized, and epoxidized [7] buckypapers.

Buckypaper	Total Cumulative Volume (mm^3^/g)	Total Specific Surface Area (m^2^/g)	Average Pore Radius (nm)	Total Porosity (%)	Bulk Density (g/cm^3^)	Apparent Density (g/cm^3^)
HG-O	605	128	12	39	0.63	1.04
MG-O	1202	186	22	53	0.44	0.92
SG-O	1661	218	31	61	0.37	0.95
HG-E	2283	192	69	74	0.32	1.26
MG-E	2116	194	56	71	0.33	1.17
SG-E	2079	218	48	69	0.33	1.08

**Table 4:** Ultimate strength (σ), strain to failure (ε) and modulus of elasticity (E) of neat, oxidized, and epoxidized [7] buckypapers from tensile experiments.

Buckypaper	σ (MPa)	ε (%)	E (GPa)
HG-O	14.0 ± 1.8	0.7 ± 0.1	2.8 ± 0.2
MG-O	6.4 ± 1.3	1.0 ± 0.1	0.9 ± 0.2
SG-O	4.2 ± 1.1	1.7 ± 0.1	0.6 ± 0.2
HG-E	2.10 ± 0.1	2.35 ± 0.32	0.20 ± 0.02
MG-E	2.87 ± 0.4	2.23 ± 0.15	0.46 ± 0.12
SG-E	3.20 ± 0.3	2.21 ± 0.17	0.60 ± 0.11

**Table 5:** Maximum flexural stress (σ), strain to failure (ε), flexural modulus (E) and estimated V_f_ of oxidized, epoxidized [7] nanocomposites, and neat resin from flexural measurements.

Material	σ (MPa)	ε (%)	E_f_ (GPa)	V_f_ (%)
HG-O-nanocomposite	171 ± 8	2.1 ± 0.4	8.38 ± 0.3	16
MG-O-nanocomposite	200 ± 4	2.9 ± 0.4	8.08 ± 0.2	14
SG-O-nanocomposite	203 ± 3	4.1 ± 0.2	6.40 ± 0.2	11
HG-E-nanocomposite	206 ± 5	7.6 ± 0.6	6.45 ± 0.1	7.5
MG-E-nanocomposite	211 ± 6	7.6 ± 0.4	6.56 ± 0.2	9
SG-E-nanocomposite	232 ± 5	7.6 ± 0.5	6.63 ± 0.2	10
Resin	136 ± 5	8.2 ± 0.5	3.44 ± 0.12	-

**Table 6:** σ’ conductivity of nanocomposites and epoxy resin.

Material	σ’ at 0.1 Hz (S/cm)
HG-E-nanocomposite	5.1 × 10^−4^
MG-E-nanocomposite	1.7 × 10^−3^
SG-E-nanocomposite	5.7 × 10^−3^
HG-O-nanocomposite	2.7 × 10^−3^
MG-O-nanocomposite	1.4 × 10^−3^
SG-O-nanocomposite	1.0 × 10^−3^
Epoxy resin	1.5 × 10^−15^
